# Some genetic differences in patients with rheumatoid arthritis

**DOI:** 10.1186/s13104-023-06559-w

**Published:** 2023-10-17

**Authors:** Hosam M. Ahmad, Zaki M. Zaki, Asmaa S. Mohamed, Amr E. Ahmed

**Affiliations:** 1 Biotechnology and Life Sciences Department, Faculty of Post Graduate Studies for Advanced Sciences, Beni-Suef University, Beni-Suef, 61511 Egypt; 2 Internal Medicine and Biomedical Chemistry Departments, Egypt Ministry of Health and Population, Minia, Egypt; 3 Clinical Pathology Department, Faculty of Medicine, Minia University, Minia, 61511 Egypt; 4 Clinical Pharmacy and Pharmacy Practice Department, Faculty of Pharmacy, Port Said University, Port Said, 42526 Egypt

**Keywords:** Gene polymorphisms, FokI, TaqI, Rheumatoid arthritis, Polymerase chain reaction, Parathyroid hormone

## Abstract

**Objective:**

Vitamin D is important for bone and cartilage metabolism. Changes in vitamin D blood level may be related to pathological disorders such as rheumatoid arthritis (RA). The main aim of this study is to investigate the association between RA and the vitamin D receptor (VDR) genes FokI and TaqI polymorphisms. One hundred RA patients and fifty healthy matched controls were assessed for VDR FokI and TaqI genotyping. Intact parathyroid hormone (PTH) and calcium (Ca) levels were measured, categorized, and compared between the cases and control groups.

**Results:**

We found that the FokI genotype frequencies for the RA cases and control groups were FF:Ff:ff = 46%:52%:2% and 50%:50%:0%, respectively (P = 0.76). The TaqI genotype frequencies for the RA cases and control groups were TT:Tt:tt = 45%:44%:11% and 42%:42%:16%, respectively (P = 0.69). A statistically significant high serum PTH level was associated with the ff genotype (p = 0.03), and a significantly low serum Ca level was associated with the TT genotype (p = 0.003). In comparison with controls, no influence of VDR FokI and TaqI genotypes on RA susceptibility or risk was demonstrated.

## Introduction

Persistent symmetric polyarthritis (synovitis) is the defining characteristic of rheumatoid arthritis (RA), a chronic multisystem inflammatory illness. Any joint can be affected, though an extra-articular association of organs such as the skin, heart, lungs, and eyes can occur. Rheumatoid arthritis (RA) affects the joints, causing them to become warm, swollen, and painful. Pain and stiffness often worsen after periods of inactivity [1].

The vitamin D receptors (VDR) act as ligand-activated, transcriptional-controlling proteins. They selectively bind the 1,25-dihydroxyvitamin D3 [1,25(OH)2D3] hormone and control the expression of selected genes in target cells [2]. Vitamin D receptor binding sites are enriched in gene loci associated with autoimmunity and RA [3]. Insufficiency of vitamin D and changes in VDR function have been related to increased vulnerability to infection, malignancy, and autoimmune diseases like RA [4].

The VDR FokI gene (rs2228570) has a unique role in immunity [5]. The FokI F allele was found to influence immune cell behavior and may be associated with altered immune function, which can result in a more active immune system [6].

The VDR TaqI gene (rs731236) resulted in a T-to-C substitution at codon 352 within exon 9 [7]. The varied TaqI genotypes are more prevalent in some populations than others, probably as a result of different evolutionary processes [7]. The VDR gene TaqI may modify the vitamin D metabolic pathway by altering the interaction between the vitamin D receptor and the active circulating vitamin D [8]. It has been demonstrated that the TT genotype is linked to reduced levels of vitamin D [7].

**Fig. 1:**
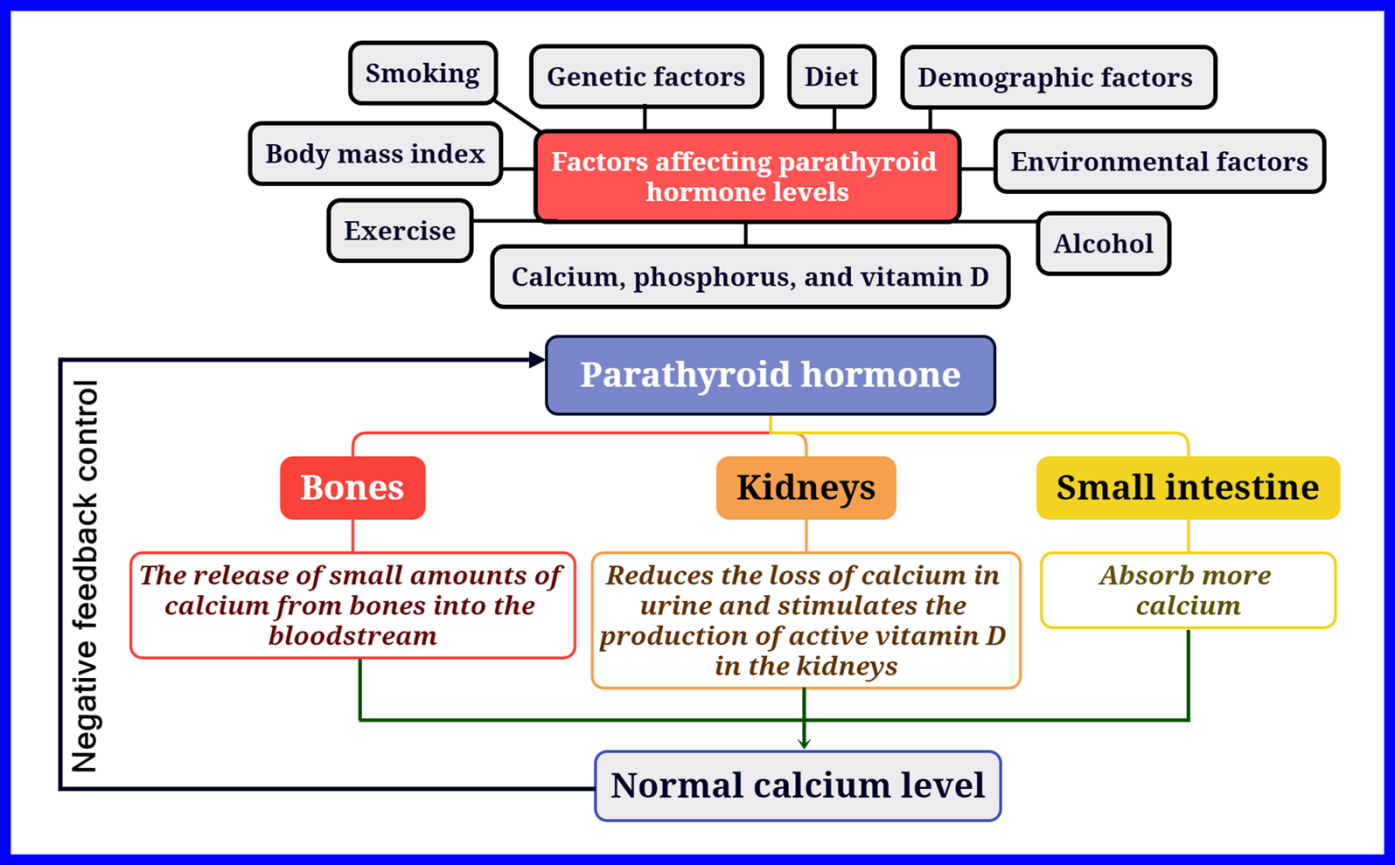
The control and action of parathyroid hormone

Parathyroid hormone (PTH) is secreted by four parathyroid glands. Normally, its production is increased when calcium (Ca) levels in the blood are low. PTH sends a signal to the bones to release Ca into the blood and to the kidneys to reabsorb Ca and excrete phosphorus. Additionally, PTH plays an important role in intestinal absorption by causing the conversion of vitamin D into its active form. PTH increases the activation of 25-hydroxy vitamin D to 1,25-dihydroxy vitamin D in the kidneys, which then motivates the intestines to absorb both calcium and phosphorus [9]. The normal reference range for intact (whole) PTH is 10–65 pg/mL [10]. The relationship between rheumatoid arthritis and parathyroid hormone is not yet fully understood due to the many factors that influence parathyroid hormone levels in the blood [11]. The control and action of parathyroid hormone are shown in Fig. 1.

The aim of this study is to compare the VDR genes FokI and TaqI in RA cases and controls and to evaluate their relationship to PTH and Ca levels.

## Methods

### Study design

This case–control study was carried out at special rheumatology clinics, where all medical assessments were performed.

### Study population

**Fig. 2:**
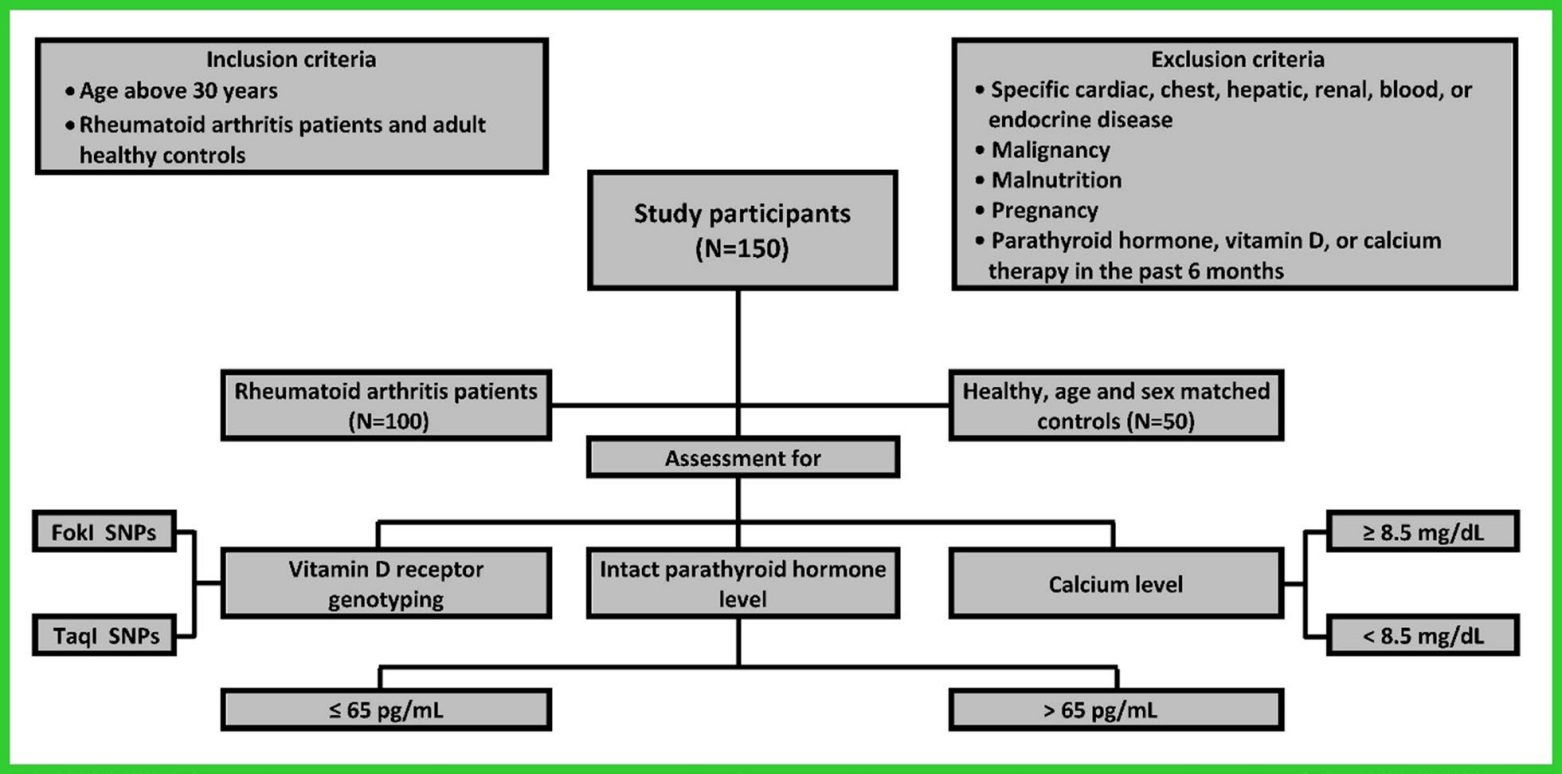
Flow diagram of the study

Adults aged over 30 years were divided into two groups: the first group (cases) included 100 previously diagnosed rheumatoid arthritis patients who met the 2010 “American College of Rheumatology/European League against Rheumatism classification criteria for RA patients”. [12]. The second group (control) included 50 healthy adults matched for age and sex with the cases. The enrollment was performed according to the inclusion and exclusion criteria (Fig. 2).

### Procedures

Samples for both the cases and control groups were selected by consecutive sampling. All patients underwent a complete history-taking, medical examination, laboratory, and radiological investigations. Figure 2 summarizes the main steps of the study.

### Outcomes and measures

For all cases and controls, we measured serum intact PTH levels, calcium (Ca), and assessed for VDR gene FokI and TaqI genotypes.

The Calbiotech intact PTH ELISA Kit was used for the quantitative determination of intact PTH in human serum or plasma. The intact PTH level normal range is 10–65 pg/mL; we categorized them as ≤ 65 pg/mL and > 65 pg/mL.

The Spinreact Kit was used for the quantitative determination of calcium in human serum or plasma. The normal calcium level range is (8.5–10.5 mg/dL); we categorized it into (≥ 8.5 mg/dL) and (< 8.5 mg/dL).

Relationships between FokI and TaqI genotypes, PTH levels, and Ca levels were compared between cases and controls.

### Genotyping

**Fig. 3:**
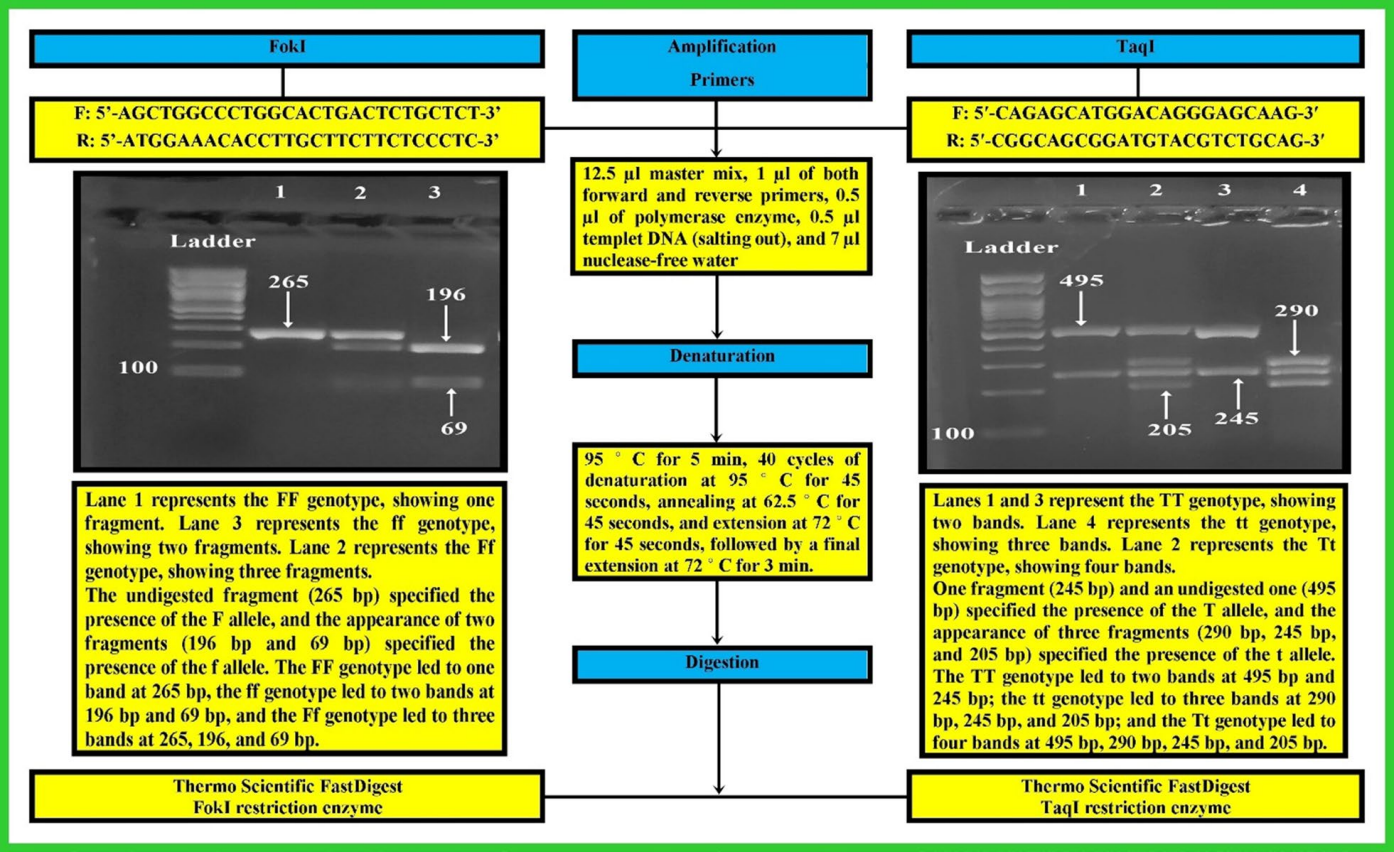
Gel electrophoresis of the PCR–RFLP technique for amplified FokI and TaqI genotypes using a 100 bp ladder

The FokI (rs2228570), and TaqI (rs731236) polymorphisms were analyzed using polymerase chain reaction—restriction fragment length polymorphism (RFLP). Genomic DNA was extracted from peripheral white blood cells using the salting out method (Fig. 3).

The study protocol was approved by the Ethics Committee of Minia University. Written informed consent was obtained from the study participants after describing the study's goals and benefits.

## Statistical analysis

Data entry and statistical analysis were performed using SPSS version 26 software. Results were presented as counts and percentages for categorical variables or means ± standard deviations (M ± SD) for continuous variables. Chi-Square and Fisher’s exact tests were used for comparing categorical data. An independent t-test was used in the case of two unrelated groups. One-way analysis of variance (ANOVA) was used to compare the means of three or more independent groups. A p-value of 0.05 was set as the threshold of statistical significance. MedCalc version 20 software was used to analyze the receiver operating characteristic (ROC) curve.

## Results

This study included one hundred rheumatoid arthritis patients and fifty healthy controls. 32 were male and 118 were female.

Table 1 shows no significant difference in gender, age, or PTH levels between the two groups (p = 0.16, 0.49, and 0.77, respectively). The M ± SD of age in cases and controls were 45.8 ± 10.01 and 44.62 ± 9.72, respectively. The M ± SD of PTH levels in cases and controls were 40.35 ± 48.9 and 38.18 ± 26.26, respectively. The M ± SD of Ca levels in cases and controls were 8.48 ± 1.17 and 9.81 ± 1.45, respectively, with a significant difference between them (p =  < 0.001).

**Table 1:** Baseline characteristics of the studied sample

	Cases N = 100	Controls N = 50	P	Total
Gender (male/female), n (%)	18/82 (18/82)%	14/36 (28/72)%	0.16	32/118 (21.3/78.7)%
Age (years), M ± SD	45.8 ± 10.01	44.62 ± 9.72	0.49	45.41 ± 9.89
PTH (pg/ ml), M ± SD	40.35 ± 48.9	38.18 ± 26.26	0.77	39.63 ± 42.68
Calcium (mg/dl), M ± SD	8.48 ± 1.17	9.81 ± 1.45	** < 0.001**	8.93 ± 1.41

**Fig. 4:**
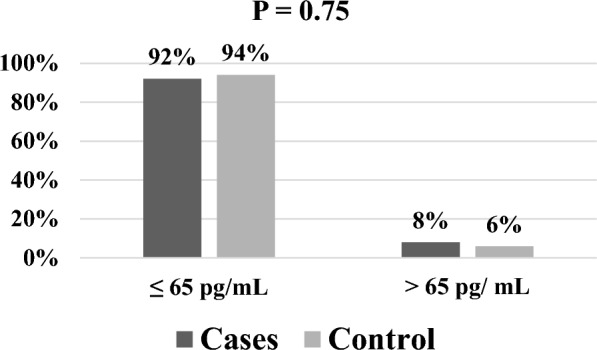
Comparison of serum PTH level categories between cases and control groups

Figure 4 shows that the percentages of PTH levels ≤ 65 pg/ml in cases and controls were 92% and 94%, respectively, with no significant difference between them (p = 0.75).

Table 2 shows the distribution of the VDR FokI and TaqI genotypes and alleles in RA patients and controls.

**Table 2:** Distribution of VDR genes FokI and TaqI genotypes in cases and controls

VDR gene	Cases N = 100		Controls N = 50		*χ*^2^	P
	**N**	**(%)**	**N**	**(%)**		
FokI genotypes						
FF	46	46	25	50	0.757	0.76
Ff	52	52	25	50		
ff	2	2	0	0		
F allele	144	72	75	75		
f allele	56	28	25	25		
TaqI genotypes						
TT	45	45	21	42	0.76	0.69
Tt	44	44	21	42		
tt	11	11	8	16		
T allele	134	67	63	63		
t allele	66	33	37	37		

FokI genotype frequencies for the RA cases and control groups were FF:Ff:ff = 46%:52%:2% and 50%:50%:0%, respectively (P = 0.76). In addition, the frequencies of the F and f alleles in RA cases were 72%:28% and 75%:25% in the control group.

TaqI genotype frequencies for the RA cases and control groups were TT:Tt:tt = 45%:44%:11% and 42%:42%:16%, respectively (P = 0.69). In addition, the frequencies of the T and t alleles in RA cases were 67%:33% and 63%:37% in the control group.

We noticed that having any of the FokI genotypes (FF, Ff, or ff) had no higher risk of being an RA patient, and likewise for TaqI genotypes (TT, Tt, or tt).

So, there is no contribution of the vitamin D receptor genes FokI and TaqI genotypes to the development of RA.

Table 3 shows that the M ± SD of the serum PTH levels in FF, Ff, and ff genotypes were 38.22 ± 44.87, 38.75 ± 49.82, and 131 ± 59.4, respectively, with a statistically significant difference among these values (p = 0.03). The M ± SD serum PTH levels between FF + Ff and ff genotypes were 38.5 ± 47.32 and 131 ± 59.4, respectively, with a statistically significant difference between these values (p = 0.008), indicating that significantly high serum PTH levels are associated with ff genotypes. The M ± SD serum PTH levels between ff + Ff and FF genotypes were 42.17 ± 52.58 and 38.22 ± 44.87, respectively, with no statistically significant difference between these values (p = 0.69).

**Table 3:** Intact parathyroid hormone and Ca levels based on the genotype distribution of the FokI gene

FokI	PTH (pg/ml)						Ca (mg/dl)
	M ± SD						M ± SD
FF	38.22 ± 44.87						8.43 ± 1.14
Ff	38.75 ± 49.82						8.56 ± 1.21
ff	131 ± 59.4						7.8 ± 0.42
P	**0.03**						0.62
	*p1*	0.96	*p2*	**0.008**	*p3*	**0.009**	
FF + Ff	38.5 ± 47.32						8.5 ± 1.17
ff	131 ± 59.4						7.8 ± 0.42
P	**0.008**						0.41
ff + Ff	42.17 ± 52.58						8.53 ± 1.19
FF	38.22 ± 44.87						8.43 ± 1.14
P	0.69						0.68

Post hoc multiple comparisons: p1 = FF vs. Ff, p2 = FF vs. ff, and p3 = Ff vs. ff

The M ± SD serum Ca levels in FF, Ff, and ff genotypes were 8.43 ± 1.14, 8.56 ± 1.21, and 7.8 ± 0.42, respectively, with no statistically significant difference among these values (p = 0.62). The M ± SD serum Ca levels between FF + Ff and ff genotypes were 8.5 ± 1.17 and 7.8 ± 0.42, respectively, with no statistically significant difference between these values (p = 0.41). The M ± SD serum Ca levels between ff + Ff and FF genotypes were 8.53 ± 1.19 and 8.43 ± 1.14, respectively, with no statistically significant difference between these values (p = 0.68).

**Fig. 5:**
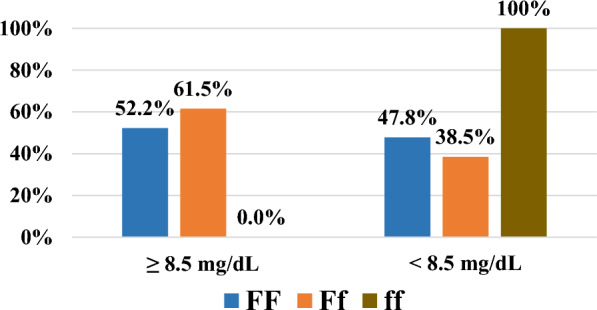
Association between the VDR gene FokI genotypes and serum Ca level categories

Figure 5 shows that the percentages of Ca levels ≥ 8.5 mg/dl for the FF, Ff, and ff genotypes were 52.2%, 61.5%, and 0%, respectively. The ff genotype was associated with low serum calcium levels, according to the calcium level categories.

Table 4 shows that the M ± SD serum PTH levels in TT, Tt, and tt genotypes were 41.4 ± 51.49, 33.91 ± 26.19, and 61.82 ± 91.88, respectively, with no statistically significant difference among these values (p = 0.24). The M ± SD serum PTH levels between TT + Tt and tt genotypes were 37.7 ± 40.92 and 61.82 ± 91.88, respectively, with no statistically significant difference between these values (p = 0.12). The M ± SD serum PTH levels between tt + Tt and TT genotypes were 39.49 ± 47.29 and 41.4 ± 51.49, respectively, with no statistically significant difference between these values (p = 0.85).

**Table 4:** Intact parathyroid hormone and Ca levels based on the genotype distribution of the TaqI gene

TaqI	PTH (pg/ml)	Ca (mg/Dl)					
	M ± SD	M ± SD					
TT	41.4 ± 51.49	8.08 ± 1.16					
Tt	33.91 ± 26.19	8.91 ± 1.15					
tt	61.82 ± 91.88	8.43 ± 0.55					
P	0.24	**0.003**					
		p1	**0.001**	p2	0.35	p3	0.198
TT + Tt	37.7 ± 40.92	8.49 ± 1.22					
tt	61.82 ± 91.88	8.43 ± 0.55					
P	0.12	0.87					
tt + Tt	39.49 ± 47.29	8.82 ± 1.07					
TT	41.4 ± 51.49	8.08 ± 1.16					
P	0.85	**0.001**					

Post hoc multiple comparisons: p1 = TT vs. Tt, p2 = TT vs. tt, and p3 = Tt vs. tt

The M ± SD serum Ca levels in TT, Tt, and tt genotypes were 8.08 ± 1.16, 8.91 ± 1.15, and 8.43 ± 0.55, respectively, with a statistically significant difference among these values (p = 0.003). Multiple comparisons revealed significantly lower serum Ca levels associated with the TT genotype than the Tt genotype (p = 0.001).

The M ± SD serum Ca levels between TT + Tt and tt genotypes were 8.49 ± 1.22 and 8.43 ± 0.55, respectively, with no statistically significant difference between these values (p = 0.87). The M ± SD serum Ca levels between tt + Tt and TT genotypes were 8.82 ± 1.07 and 8.08 ± 1.16, respectively, with a statistically significant difference between these values (p = 0.001), Thus significantly low serum Ca levels were associated with the TT genotype.

**Fig. 6:**
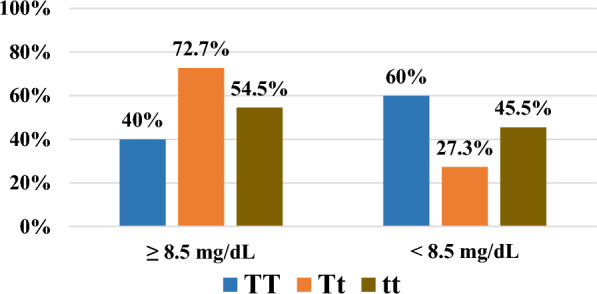
Association between TaqI genotypes and serum Ca level categories

Figure 6 shows that the percentages of Ca levels ≥ 8.5 mg/dl in the TT, Tt, and tt genotypes were 40%, 72.7%, and 54.5%, respectively. The TT genotype was associated with low serum calcium levels, according to the calcium level categories.

**Fig. 7:**
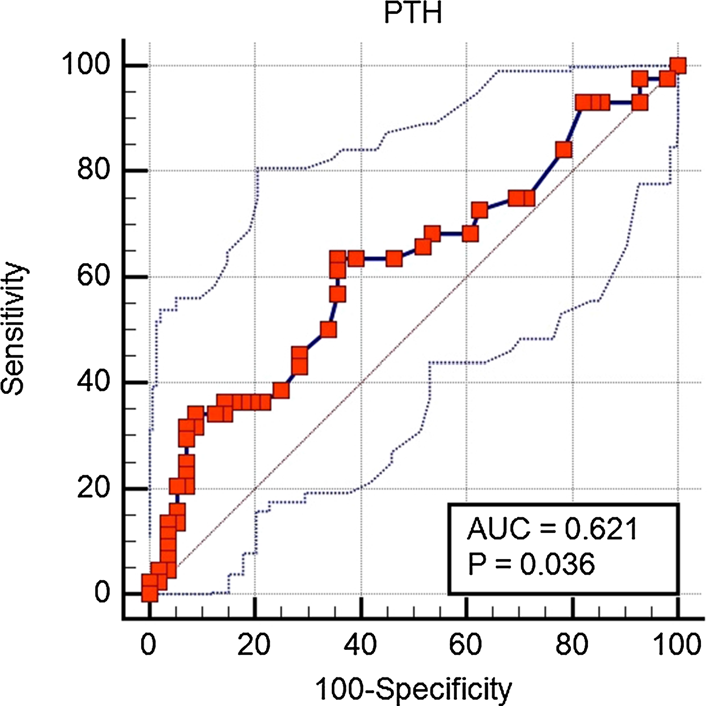
Analysis of the ROC curve for PTH level as a predictor of hypocalcemia in RA patients

Figure 7 shows that the level of serum PTH is effective in predicting hypocalcemia based on the ROC curve and its area under the curve. The accuracy, sensitivity, specificity, positive predictive value (PPV), and negative predictive value (NPV) were 28%, 64.3%, 63.6%, 69.2%, and 58.3%, respectively, with an AUC of 0.62 (p = 0.036) and a cut-off point of 28 ng/ml. From this figure, we can conclude that the level of serum PTH can predict hypocalcemia and may be a good predictive factor in controlling calcium levels in RA patients.

## Discussion

In this study, we compared RA patients and a healthy control group regarding the vitamin D receptor genes FokI and TaqI polymorphisms, PTH level, and Ca level. FokI and TaqI genotypes were detected using the PCR–RFLP technique, and the results showed that there were no significant differences in genotype or allele frequency observed between the two groups. This suggests that there is no association between the FokI and TaqI genes and the prevalence of RA disease.

Many previous studies have supported these findings. A study in the Lithuanian population found that none of the four VDR gene polymorphisms (BsmI, FokI, ApaI, and TaqI) were associated with a genetic predisposition to RA. [13]. An Indian study also found that the pattern of genotype and allele distribution in the RA disease and control groups suggested a lack of association between VDR FokI and RA susceptibility [14]. Another Korean study concluded that VDR gene alleles seem not to be associated with bone erosion in RA patients [15]. Consistent with these findings, a case–control study in the German population found no evidence of an association between RA and VDR genes [16]. A study concluded that there was no association detected for VDR ApaI and TaqI genotypes with RA risk (P > 0.05). TaqI and ApaI polymorphisms may be involved in Behcet’s disease (BD) pathogenesis. They may be possible markers in BD more than susceptibility genes. TaqI and ApaI polymorphisms appeared not to be concerned with RA pathogenesis [17].

In contrast to previous studies, a case–control study was conducted to test for polymorphisms associated with RA (VDR ApaI, BsmI, FokI, and TaqI genes) and other genes by genotyping 105 RA patients and 80 controls. The conclusion was that all the studied single nucleotide polymorphisms (SNPs) may contribute to the susceptibility of RA disease, except for the FokI SNP [18]. Another study demonstrated that the alleles of TaqI, BsmI, and FokI genotypes were associated with RA susceptibility in the total population. Ethnicity showed that BsmI variants among Africans and FokI variants among Asians and Caucasians had a significantly higher risk of RA. ApaI genotypes and RA risk have not been linked yet. These data may provide information that could lead to the development of biomarkers for RA risk [19]. Studies on RA patients have revealed that VDR polymorphisms are linked to the development of RA. The osteoporosis in RA is affected by the vitamin D receptor gene BsmI [20]. Regarding the VDR FokI, the F allele and F/F genotype are associated with RA in Europeans [21].

In this study, we found no statistically significant difference between RA cases and control groups regarding age, gender, and PTH level, but a statistically significant difference was found between the two groups regarding Ca level (p < 0.001).

The relationship between rheumatoid arthritis (RA) and parathyroid hormone (PTH) levels is ambiguous and unclear. Therefore, the role of RA disease in PTH levels is not the main aim or focus of this study. Many patients with active RA exhibited biochemical features suggestive of hyperparathyroidism, despite having normal serum PTH levels [22]. PTH and calcitonin levels can be influenced by a variety of factors, including genetic factors [23], demographic factors like age [24], gender [25], and environmental factors [26]. Genetic factors are estimated to explain 60% of the variation in PTH levels [11]. Increased serum levels of vitamin D and calcium suppress PTH secretion, while increased serum phosphate levels stimulate PTH secretion [27]. In addition to blood levels of calcium, phosphorus, and vitamin D, many other factors can influence PTH levels, including smoking, body mass index (BMI), diet and specific macronutrients, alcohol, and exercise [11].

Agreeing with these results, a study revealed that serum PTH levels were in the normal range in 81.6% of patients with active RA and in 88.7% of patients with silent RA (p = 0.331). The serum level of PTH was also not affected by patients’ gender, age, or duration of the disease [28]. Another study revealed that neither active RA nor glucocorticoid therapy appear to induce secondary hyperparathyroidism [29].

In contrast to these findings, a study found that the presence of bone erosion in RA is associated with low bone mineral density levels and high PTH levels. These associations in people with RA are independent of other common factors of bone mass and mineral metabolism and the severity of functional impairment [30].

We found a significant difference between the RA and control groups regarding Ca level (p < 0.001) in this study. In a cross-sectional study of 394 patients with rheumatoid arthritis investigating the total serum calcium levels, Ca levels were lower in RA than in healthy adults [31]. Calcium and phosphorus metabolism are altered in RA, with a decrease in the serum calcium-to-phosphorus ratio. In RA cases, the serum calcium-to-phosphorus ratio was 1.51 ± 0.35, compared to 2.85 ± 0.5 in controls (p < 0.001) [32]. A study revealed a significantly decreased calcium to phosphorus ratio in RA patients compared to controls, which obviously shows that there is a change in calcium and phosphorous metabolism in RA. As calcium and phosphorus are essential constituents of bone, bone metabolism is ultimately altered in rheumatoid arthritis [33, 34].

In this study, patients with the ff genotype had significantly high serum PTH levels. However, in a cross-sectional study of 50 hemodialysis patients and 30 healthy adults as a control group, the FF genotype of the FokI gene was more frequent in hemodialysis patients with higher intact PTH levels [35].

Against the study results, another study found that between the VDR genotypes determined by PCR, there was no detectable variation in serum PTH levels for diabetic patients in a study analyzed by PCR–RFLP of the VDR gene in 877 Japanese hemodialysis patients [36]. Also, the variant alleles of the BsmI and FokI genes were not linked to the emergence of renal disease or secondary hyperparathyroidism among a sample of Egyptian patients on maintenance hemodialysis [37].

## Limitations

This study has some limitations. First, the results cannot be generalized to all populations because the study was limited to participants from one country without including different ethnicities. Similar studies are needed in multiple countries to generalize the results. Second, the study biomarkers were assessed only once, so longitudinal data are not available.

## Conclusion

There is no contribution from the vitamin D receptor genes FokI and TaqI genotypes to the development of RA. Parathyroid hormone levels are not affected by RA disease; on the contrary, blood Ca levels decrease in RA patients. Rheumatoid arthritis may cause hypocalcemia but may not affect PTH secretion because of other controlling factors.

Regarding FokI and TaqI genotypes, significantly high serum PTH levels are associated with ff genotypes and significantly low serum Ca levels are associated with TT genotypes. There is a possible role of FokI and TaqI genes in controlling the levels of PTH hormone and calcium. Intact serum PTH levels were effective in predicting hypocalcemia in RA patients.

## Data Availability

The datasets generated and/or analyzed in this study are available from the corresponding author on reasonable request.

## Abbreviations

VDR: Vitamin D receptor
RA: Rheumatoid arthritis
PTH: Intact parathyroid hormone
Ca: Calcium
PCR: Polymerase chain reaction
RFLP: Restriction fragment length polymorphisms
